# The Role of Ultrasound in Screening Dense Breasts—A Review of the Literature and Practical Solutions for Implementation

**DOI:** 10.3390/diagnostics8010020

**Published:** 2018-03-16

**Authors:** Denise Thigpen, Amanda Kappler, Rachel Brem

**Affiliations:** Department of Radiology, The George Washington University, Washington, DC 20037, USA; akappler@mfa.gwu.edu (A.K.); rbrem@mfa.gwu.edu (R.B.)

**Keywords:** screening breast ultrasound, breast cancer, breast density, automated breast ultrasound, mammography

## Abstract

Breast cancer is the second leading cause of cancer death in women. Estimates indicate a nearly 40% breast cancer mortality reduction when screening women annually starting at age 40. Although mammography is well known to be a powerful screening tool in the detection of early breast cancer, it is imperfect, particularly for women with dense breasts. In women with dense breast tissue, the sensitivity of mammography is reduced. Additionally, women with dense breasts have an increased risk of developing breast cancer while mammography has a lower sensitivity. Screening ultrasound, both handheld and automated, is effective in detecting mammographically occult cancer in women with dense tissue. Studies have shown that ultrasound significantly increases detection of clinically important, small, largely invasive, node-negative cancers. The purpose of this review article is to summarize the literature to date regarding screening breast ultrasound, emphasizing differences in cancer detection in high risk and intermediate risk women, and to discuss practical ways to implement screening ultrasound in clinical practice, including automated whole breast ultrasound, as a viable solution to the increasing need for additional screening.

## 1. Introduction

In the United States, an estimated 252,710 new cases of invasive breast cancer will have been diagnosed in 2017 and 40,610 women will have died of their disease. Breast cancer is the second leading cause of cancer death in women [1]. It is well established that early detection reduces breast cancer deaths [2]. Estimates indicate a nearly 40% breast cancer mortality reduction when screening women annually starting at age 40 [3]. 

**Figure 1:**
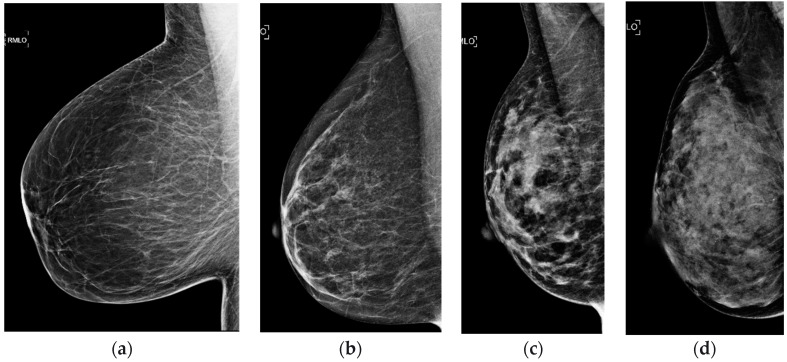
Breast density (**a**) almost entirely fatty, (**b**) scattered fibroglandular tissue, (**c**) heterogeneously dense, and (**d**) extremely dense, as determined by the BI-RADS Atlas [4].

Although mammography is well known to be a powerful screening tool in the detection of early breast cancer, it is imperfect, particularly for women with dense breasts. Breast density refers to the relative amounts of fat and glandular tissue in the breast. This ranges from nearly all fat to nearly all glandular tissue and affects the mammographic appearance of the breast (Figure 1). Breast density description has been standardized by the American College of Radiology (ACR) Breast Imaging Reporting and Data System (BI-RADS) Atlas [4]. The descriptions of these categories have changed over the years. The 5th Edition, published in 2013, emphasizes the text descriptions of density and also allows for the tissue composition categories to be referred to as a–d, while the 4th Edition, published in 2003, emphasized the numeric percentage of dense tissue and referred to the categories as 1–4 (Table 1) [5]. For the purposes of this article, all density classifications will be referred to by their text description or by the 5th edition categories a–d. Conventionally, the two least dense categories (fatty and scattered) are referred to as “non-dense” and the two most dense categories (heterogeneously dense and extremely dense) are referred to as “dense.” 

Overall, the sensitivity of mammography for the detection of breast cancer is 85%; however, in women with dense breast tissue, the sensitivity of mammography is reduced to 47.8–64.4% [6]. Not only is mammography less sensitive in women with dense breasts, women with extremely dense breasts have a 4.7-fold increased risk of developing breast cancer [7]. Therefore, women with dense breasts have a higher risk of breast cancer, yet mammography is less effective. Cancers detected in women with dense breasts are larger and more often node positive [8]. Interval cancers, which have a worse prognosis than screen-detected cancers, are 18 times more likely to occur in women with dense breasts [7]. This is even more significant since more than half of American women have dense breast tissue [9]. Given the prevalence of dense breast tissue and the challenges of identifying cancer in dense breasts with mammography, additional imaging modalities to detect mammographically occult breast cancer are needed. 

As of January 2018 in the United States, 30 states have “density notification” laws requiring women to be informed of their breast density, many mandating that women be informed that additional screening can detect cancer not visible with mammography [10]. The issue of dense breast tissue and its impact on both breast cancer risk and mammographic limitation is increasingly being featured in the lay press and media. The concept of individualized, risk-based screening is increasingly taking hold. 

At present, there is a risk-stratified screening model in place in the United States. Mammography is the mainstay of screening for women aged 40 and over. High-risk populations (women with a lifetime risk of greater than 20–25%) are advised to undergo additional annual surveillance with magnetic resonance imaging (MRI) [11,12,13] or if they cannot undergo MRI, the ACR now recommends they should consider screening breast ultrasound (SBU) [14]. However, there is a gap in the approach to screening intermediate risk women (women with a lifetime risk of 15–20%), who may not qualify for high risk screening with MRI. Women with dense breasts constitute the largest portion of this intermediate group and stand to benefit from early detection using adjunct screening approaches in addition to screening mammography.

**Figure 2:**
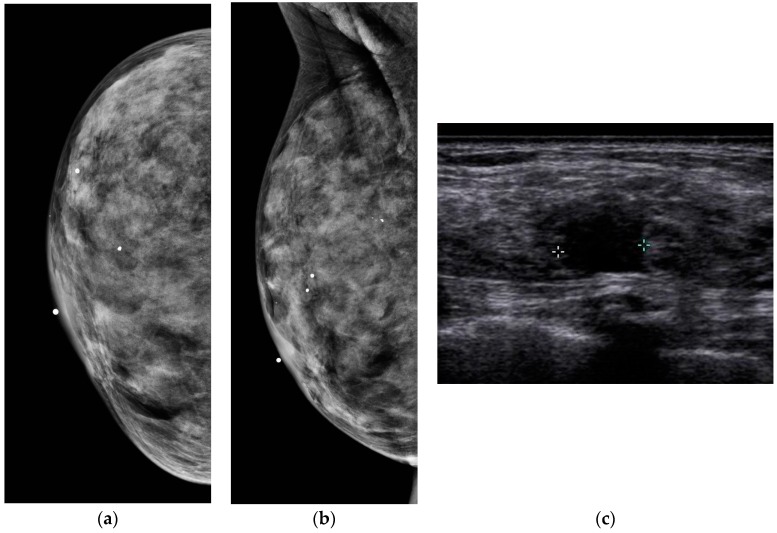
A 43-year-old woman with extremely dense breast tissue. Dense tissue obscures a breast cancer that is easily visible with ultrasound imaging. (**a**,**b**) Craniocaudal (CC) and mediolateral oblique (MLO) digital mammography. (**c**) Handheld high-resolution ultrasound demonstrates a 1.2 cm irregular mass, denoted by calipers in the image, which was biopsied and proven to be invasive ductal carcinoma.

Dense breast tissue appears white on mammograms, as does breast cancer, which is why dense tissue can sometimes obscure a cancer. In contrast, dense tissue is echogenic on ultrasound, while breast cancer is hypoechoic. Ultrasound leverages the differences in tissue characteristics to improve cancer detection in women with dense breasts (Figure 2).

**Figure 3:**
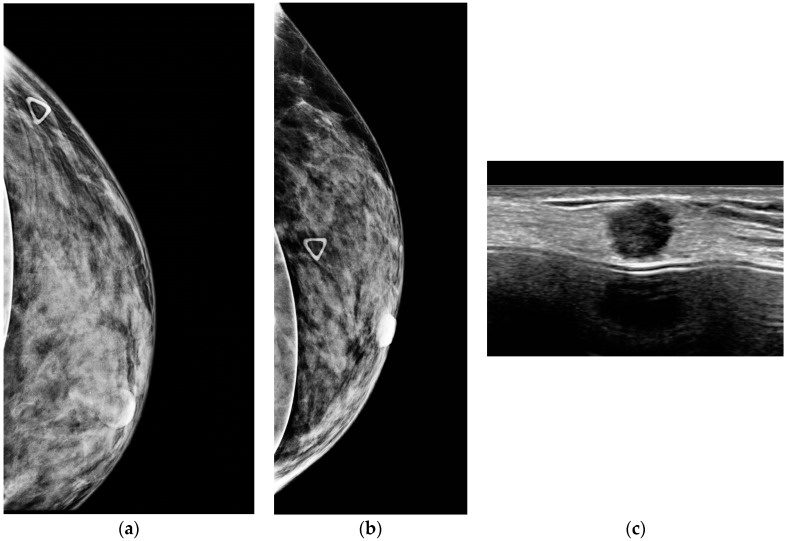
A 53-year-old woman with dense breasts and a palpable abnormality presents for evaluation. (**a**,**b**) Implant displaced CC and ML digital mammograms fail to reveal a mass (triangle denotes palpable abnormality). (**c**) High resolution handheld ultrasound easily depicts a 0.9 cm spiculated mass due to invasive ductal carcinoma.

**Figure 4:**
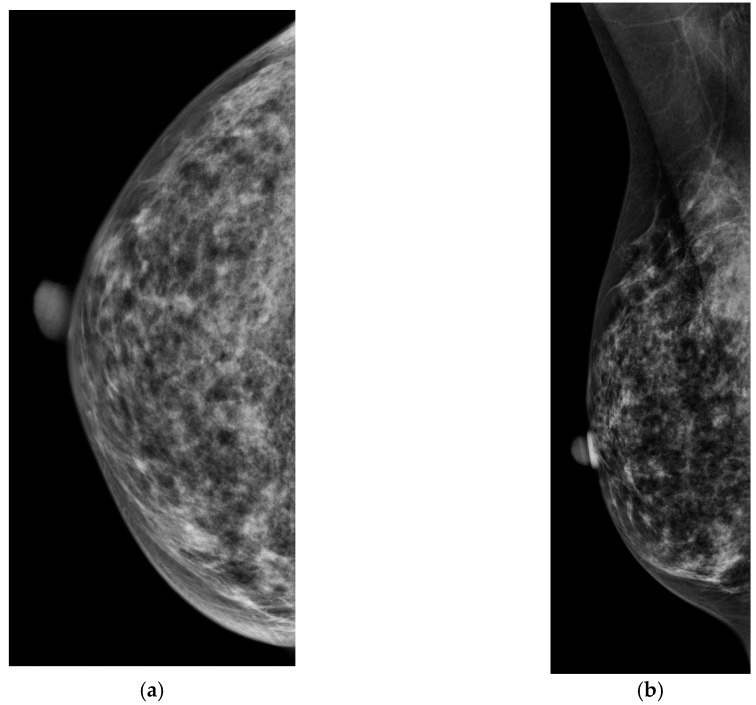

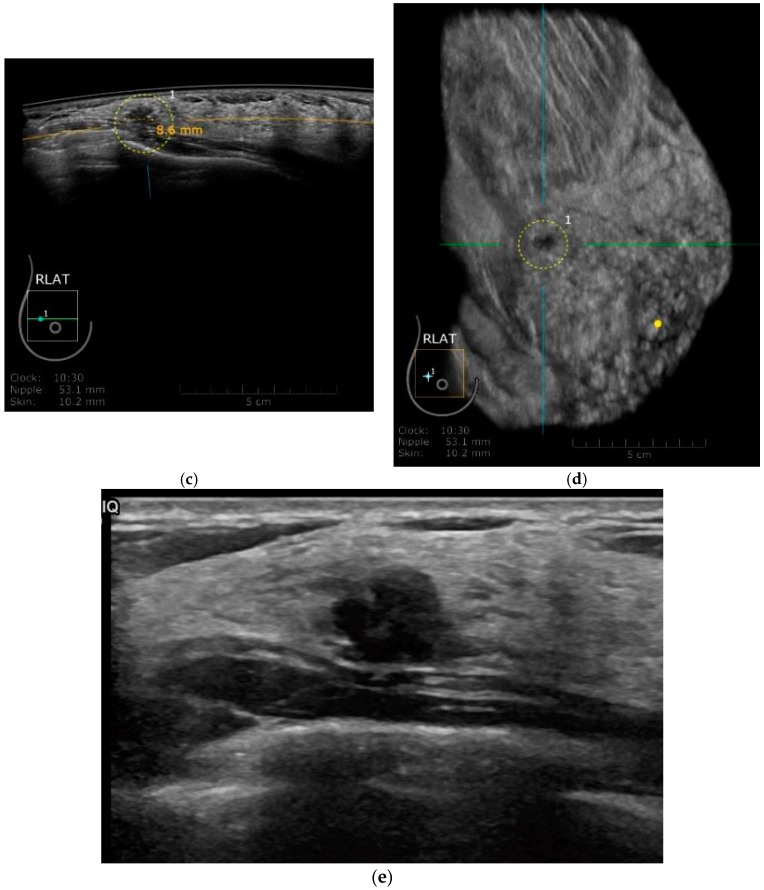
(**a**,**b**) CC and MLO digital mammograms in a 56 year old woman with heterogenously dense breasts with an occult breast cancer. Automated whole breast ultrasound (ABUS). (**c**) transverse image of ABUS and (**d**) reconstructed coronal image demonstrate an irregular hypoehoic mass. (**e**) Handheld ultrasound confirms a 0.8 cm irregular, heterogenous hypoechoic mass. Pathology demonstrated mixed invasive ductal and invasive lobular carcinoma, grade 2, ER positive, PR and HER2 negative.

Screening ultrasound, both handheld (Figure 3) and automated (Figure 4), is effective in detecting mammographically occult cancer in women with dense tissue. Studies have shown that ultrasound significantly increases detection of clinically important, small, invasive, node-negative cancers. The purpose of this review article is to summarize the literature to date regarding screening breast ultrasound (SBU), emphasizing differences in cancer detection in high risk and intermediate risk women, and to discuss practical ways to implement screening ultrasound in clinical practice, including automated whole breast ultrasound (ABUS), as a viable solution to the increasing need for additional screening.

## 2. Review of the Literature—Handheld Screening Breast Ultrasound

When analyzing the body of literature regarding screening breast ultrasound, it is paramount to evaluate the patient population studied. The incremental cancer detection of screening breast ultrasound in a high-risk population (>20% lifetime risk of cancer) will undoubtedly be higher than that found in an intermediate risk population. This difference is due to higher prevalence of occult breast cancer in high risk populations than in women with lower risk of the disease. Here, we will review landmark trials (Table 2) demonstrating the feasibility and utility of screening breast ultrasound, which populations it is most useful for, and how it can be integrated into practical screening programs.

In a single institution study in 2001, Kaplan and colleagues evaluated the performance of screening ultrasound in patients with heterogeneously dense or extremely dense BI-RADS categories with negative findings at clinical examination and negative mammography results [15]. In this study which included 1862 women, 57 biopsies were recommended in 56 patients with six breast cancers detected. This resulted in a diagnostic yield of three additional cancers per 1000 women. Notably, the sonographically detected cancers were mostly small, invasive, early stage cancers with mean size of 9 mm, all stage 0 or 1. In this study, technologists with experience in breast ultrasound performed the examination with the average time to perform the examination approximately 10 min.

In 2003, Leconte et al. compared the sensitivities of mammography with subsequent sonography for the detection of non-palpable breast cancers in patients with non-dense tissue (almost entirely fatty and scattered fibroglandular tissue) versus dense tissue (heterogeneously and extremely dense). In patients with non-dense tissue, the sensitivities of mammography and sonography were 80% and 88% respectively and this difference was not statistically significant [16]. In patients with dense tissue, however, the sensitivities were 56% for mammography and 88% for mammography plus ultrasound, a statistically significant finding, thereby determining the group for which SBU was most beneficial—women with dense breasts.

Screening breast ultrasound had been shown to find additional cancers in women with dense breasts. But how effective could it be for a screening program? Interval cancer rates can be used as a metric for assessing the effectiveness of a screening program. In 2011, Corsetti, et al. reported that adding SBU brought the interval cancer rate in dense breasted patients down to a similar level as non-dense patients, suggesting an improved screening benefit and paving the way for largescale randomized trials [29].

The initial randomized multi-center trial investigating the utility of screening breast ultrasound was the American College of Radiology Imaging Network (ACRIN) 6666 trial. This trial investigated the increase in cancer detection using handheld SBU in high risk women with dense tissue in at least one quadrant of the breast. The results of the first year were published in 2008 and demonstrated that the addition of ultrasound to screening mammography detected an additional 4.2 cancers per 1000 patients than were detected by mammography alone [17]. What is important to understand about this study was that the patients were not only dense, but they were also high risk, with at least one additional risk factor: elevated risk (lifetime risk ≥25% as assessed by either the Gail or Claus model), 5-year Gail model risk ≥2.5% or ≥1.7% and extremely dense breasts, personal history of breast cancer, prior atypical breast biopsy, history of chest, mediastinal, or axillary adenopathy, and/or BRCA1/2 mutations. There was, however, a decrease in specificity from 96% with mammography alone to 89% with mammography plus ultrasound. Notably, ultrasound examinations were performed by radiologists and took an average of 19 min.

In 2012, Berg and colleagues reported years 2 and 3 follow up mammography and ultrasound screening findings of the ACRIN 6666 trial. In years 2 and 3, an additional 3.7 cancers were detected with screening breast ultrasound per 1000 women screened [18]. The sensitivity of mammography combined with ultrasound was higher than that for mammography alone (76% vs. 52%). Importantly, the specificity of combined screening increased from 74% in the first year to 84% in years 2–3, while maintaining a similar cancer detection rate. 

Studies confirmed that the addition of ultrasound to mammography in women with increased breast density as well as increased risk of cancer resulted in a substantial increase in the detection of mammographically occult breast cancer. However, the question as to the impact of screening breast ultrasound in women with dense breast tissue without requiring additional risk factors remained. This was answered as states began implementing breast density notification laws with the increasing use of adjunct screening ultrasound in asymptomatic women with normal mammograms and dense breast tissue. 

Connecticut became the first state to pass a “density notification” law in 2009 requiring physicians to advise women of their breast density. Following the implementation of breast density reporting laws, Weigert reported the incremental cancer detection rate in all women who accepted SBU with dense breasts. In 2012, initial results of ultrasound screening in 12 practices in Connecticut which included 72,030 screening mammograms and 8647 screening ultrasound examinations over a one-year period were reported, which Weigert termed “The Connecticut Experiment”. In women with >50% breast density, the addition of screening breast ultrasound yielded an additional 3.25 breast cancers per 1000 women screened [20]. 

Similar to Weigert, Hooley and colleagues reported on their first year experience in Connecticut after the implantation of the density reporting law. In this study, 935 women were included, a majority either intermediate or low risk (81.6%), and all of which had heterogeneously dense or extremely dense breast tissue. Technologist performed handheld SBU yielded a cancer detection rate of 3.2 cancers per 1000 women screened [19]. Notably, both Weigert and Hooley had similar results despite different practice settings, the former a private practice and the latter at an academic institution.

In 2015, Weigert reported on the second year of the Connecticut Experiments. In this study, the addition of screening ultrasound in women with mammographically normal but dense breasts continued to improve breast cancer detection by finding an additional 2.3 cancers per 1000 women screened. If high risk lesions are included, a total of 3.8 cancers/high risk lesions per 1000 women screened were detected [21]. After the fourth year of the Connecticut Experiments, Weigert reported that the positive predictive value (PPV) had doubled (from 7.3% in year 1 to 20.1% in year 4) while maintaining a stable rate of cancer/high risk lesions [27]. These results suggest there is a learning curve in determining which lesions to biopsy, which can be refined with clinical experience while maintaining a lesion detection rate similar to other published studies. 

In 2016, Tagliafico and colleagues reported the interim results of a prospective screening trial Adjunct Screening with Tomosynthesis or Ultrasound in Women with Mammography-Negative Dense Breasts (ASTOUND). The goal of this study was to compare incremental breast cancer detection by tomosynthesis and handheld physician performed ultrasound in mammographically negative dense breasts. In this study which included 3231 screening participants, 24 additional breast cancers were detected, the incremental cancer detection rate for tomosynthesis detected breast cancers versus ultrasound detected breast cancers was found to be 4.0 per 1000 screens versus 7.1 per 1000 screens respectively [23]. In this study, the false positive recall rate did not differ between tomosynthesis and ultrasound. Tomosynthesis has enjoyed widespread adoption with a similar recall rate, and yet, these results demonstrate SBU outperforms tomosynthesis in incremental cancer detection rates. 

In 2017, Destounis confirmed the continued success of their screening ultrasound program by retrospectively reviewing 5434 screening ultrasounds performed on 4898 women with heterogeneously or extremely dense tissue. 95.7% of these screening exams resulted in BI-RADS 1 or 2 designations, with a postitive predictive value of 18%, an overall biopsy rate of 2.0% and an additional cancer detection rate of 3.3 per 1000. This study utilized handheld ultrasound and detected mostly small, node-negative cancers. Notable, Destounis offered screening breast ultrasound to all women with dense breasts but noted that many patients who chose to undergo additional screening had additional self-reported high risk factors [28]. This may reflect that women who perceive themselves as high-risk, will opt for additional screening, emphasizing the need for a personalized approach.

While there is ample literature demonstrating the improved cancer detection rate with the addition of SBU, there is no empirical evidence that SBU reduces breast cancer mortality [30]. In general, analysis of mortality requires long-term study. Typically, shorter-term studies use surrogate endpoints such as stage to determine benefits to the population. The Japan Strategic Anti-Cancer Randomized Trial (J-START) is the world’s first large-scale randomized controlled trial investigating the efficacy of SBU in addition to mammography in 72,998 healthy Japanese women ages 40–49, which aims to determine effects on mortality [24]. Initial results reported by Ohuchi in 2016 demonstrate that the addition of SBU increased sensitivity (91.1% compared to 77.0% for mammography alone), decreased specificity (87.7% compared to 91.4% for mammography alone) and increased cancer detection rate (5.0/1000 compared to 3.2/1000 for mammography alone) for an additional yield of 1.8 cancers/1000 women screened, with cancers more frequently stage 0 and 1 [24]. It is important to note that this trial included women of all breast densities and only women aged 40–49. Enrolling a relatively young cohort will allow long-term follow-up, but will also likely have fewer cancers detected initially, due to their young age. Although it remains to be seen how results from this population may be applied to other cohorts, further reports from this trial looking at specific density groups will be important, and mortality results from this trial may eventually influence recommendations regarding combined screening approaches with SBU.

## 3. Review of the Literature—Automated Screening Breast Ultrasound and Integrating SBU into Clinical Practice

Although studies have shown screening breast ultrasound in women with dense breast tissue to be effective in detecting mammographically occult predominantly small node- negative breast cancer, several barriers exist limiting its implementation as a screening modality. Handheld screening ultrasound, requires a great deal of resources to screen large numbers of women as the scanning is performed by the technologist. Furthermore, the identification of the sonographically detected abnormality is made by the technologist. Furthermore, the time, required for a bilateral handheld whole breast ultrasound, can range from 10 to nearly 20 min, making it challenging to implement in a clinical practice [15]. 

Additionally, handheld ultrasound is well-known to be operator dependent, and if a technologist is performing the exam, the radiologist must rely only on the representative images obtained by the technologist. Automated whole breast ultrasound allows for uncoupling of image acquisition from interpretation. The entirety of the breast can be imaged and subsequently the entire data set can be reviewed by the radiologist. This allows for more reliable and reproducible imaging of the entirety of the breast, more extensive images for annual comparison, and allows the radiologist to interpret the entire data set as opposed to representative images obtained by a technologist. Image acquisition for ABUS takes 60 s per view with a total exam time of about 15 min. Study interpretation time, performed by the radiologist is only 2.9 min [22]. Of note interpretation time does not include the time to compare to prior examinations or to generate a report. 

In a large cohort study of 1886 women, Vourtsis and colleagues found ABUS to be comparable to hand-held ultrasound; the overall agreement between the two modalities was found to be 99.8%. Moreover, ABUS seemed to outperform hand held ultrasound in the detection of architectural distortion, particularly with the use of the coronal plane [31].

Implementation of ABUS, too, has barriers. The cost of the machine (which is similar to the cost of a new handheld ultrasound unit) and dedicated workstation, along with relatively low reimbursement has thwarted its widespread adoption. Medicare reimbursement for bilateral whole breast ultrasound averages around $165 [32]. Currently, a dedicated workstation is required for viewing ABUS datasets, although future picture archiving and communications systems (PACS) developments could allow for seamless integration. Finally, with the addition of any new modality, there are there is required training time and expense for both physicians and technologist. 

Several studies to date have investigated the use of automated breast ultrasound in women with dense breasts. In 2014, Brem and colleagues in a multi-center observational study of 15,318 women with heterogeneously dense or extremely dense breast tissue demonstrated an increased detection of 1.9 cancers per 1000 screening examinations with the use of supplemental ABUS regardless of further risk characterization [22]. Moreover, of the additional cancers detected by ABUS, 93.3% were invasive and node-negative cancers, suggesting this technology detects clinically significant cancers. 

In 2016, Giger and colleagues in a reader study set out to assess and compare radiologists’ performance in the detection of breast cancer using mammography alone versus mammography with ABUS [25]. In this study, all patients were asymptomatic with heterogeneously dense or extremely dense breast tissue. The analysis included 185 cases with 133 non-cancers and 52 biopsy proven cancers. Readers first interpreted mammography alone and subsequently mammography with ABUS. Performance was compared in terms of area under the curve (AUC) for the receiver operating characteristics (ROC) curve, sensitivity and specificity. For mammographically negative cancers, mammography with ABUS yielded a statistically significant 25% relative improvement in the AUC. Notably, this study also demonstrated overall clinically insignificant decrease in specificity of 78.1% for mammography alone and 76.1% for mammography with ABUS. In 2012, the Food and Drug Administration approved automated whole breast ultrasound for use as supplemental screening in women with heterogeneously and extremely dense breasts [33].

While improving cancer detection rate of clinically important cancers, the early trials utilizing automated ultrasound as an adjunct to mammography demonstrated an increase in recall rate and a decrease in specificity (−13.4% in the SomoInsight Study) [22]. These were expected results with the addition of a new modality. With continued clinical experience, as well as subsequent rounds of screening, these parameters are expected to improve. Indeed, when using automated ultrasound combined with mammography, Wilczek reported in 2016 only 9 additional recalls per 1000 women screened and a decrease in specificity of only −0.7%, while still maintaining an additional 2.4 cancers found per 1000 women screened with combined automated ultrasound and mammography [26]. This is similar to the improvement in specificity using hand held SBU over the 4 years of the Connecticut Experiments [19].

The addition of computer-aided detection (CAD) software for ABUS is a newer technology which has the potential to improve the screening performance of radiologists. Jan C.M. van Zelst and colleagues investigated the effect of CAD software for ABUS on reading time, sensitivity, specificity and positive predictive value for eight radiologists [34]. With the addition of CAD, average reading time was significantly shorter (133.4 s/case CAD-ABUS vs. 158.3 s/case ABUS). On average, sensitivity of CAD based ABUS was similar to that of unaided conventional ABUS reading (84% for each). Although not statistically significant, the average specificity increased from 67% to 71% with the addition of CAD. Although this study showed CAD software for ABUS has the potential to improve efficiency of reading ABUS, more research is necessary to investigate the effect of CAD on breast cancer detection and recall rate in a screening program. [34].

When implementing a SBU program into a practice, one has to determine *when* to offer the exam. First, a woman’s density must be evaluated to determine if she should be offered the exam. For the patient, the ideal time to have the exam would likely be on the same day she receives her screening mammogram. Should a practice devote resources and manpower to determining her density on the spot in order to offer her additional SBU on the same day? Cohen and Margolies assessed the efficiency of using prior mammogram density information to allow discussion of supplemental SBU *before* the mammogram. They found that 81.4–90.9% of patients would be correctly counseled on their density using a mammogram result from the last 3 years, thereby reducing the barriers of additional appointments, wait-times and anxiety [35].

Finally, a concern many practices may face is how to manage the volume of additional studies for women with dense breasts. Studies have demonstrated that only 30% of patients offered SBU avail themselves of supplemental screening [27]. This allows practices to slowly learn how to incorporate breast ultrasound and by leveraging the workflow advantages offered by ABUS, many practices will likely be able to incorporate the volume of the densely breasted population. The radiology community has an opportunity to advance supplemental screening which will allow for the detection of earlier cancers in women with dense breasts, while not being overwhelmed by the learning curve.

## 4. Conclusions

In summary, women with dense breasts suffer from an increased risk of breast cancer combined with decreased sensitivity of mammography alone. Adding ultrasound screening can increase breast cancer detection rates by 1.9–4.2%, depending on the population. Automated ultrasound devices can mitigate the challenges posed by handheld screening programs, namely with faster scan times, reduced operator dependence, and improved workflow and datasets. Automated screening ultrasound, however, also has barriers to implementation, including the need for additional training, cost of the device, and possible integration into pre-existing PACS. Continued experience with this modality, however, demonstrates acceptable recall rate and sensitivity while maintaining improved cancer detection rates of clinically important cancers. 

## Figures and Tables

**Table 1:** Tissue composition descriptions used in the BI-RADS Atlas [4].

Tissue Composition	4th Edition	5th Edition
Almost entirely fatty	1	a
Scattered fibroglandular tissues	2	b
Heterogeneously dense	3	c
Extremely dense	4	d

**Table 2:** Summary of Findings in Reviewed Literature.

Study	Study Description	Method	No. of Screening US Exam	No. of US-Only Cancers	Mammography Plus Ultrasound			Additional Cancer Yield from US per 1000 Women Screened
					Sensitivity (%)	Specificity (%)	Positive Predictive Value (%)	
Kaplan [15]	BI-RADS c-d density; patients with negative clinical examination and mammographic findings; in patients with focal abnormal mammographic findings or palpable abnormalities, all areas of the breast outside of the quadrant with abnormalities were evaluated with ultrasound	Tech HHUS	1862	6	-	-	-	3.2
Kolb et al. [6]	BI-RADS b-d density; patients with no clinical symptoms	MD HHUS	13,547	37	97.3	-	-	2.73
Leconte et al. [16]	BI-RADS a-d density; palpable abnormalities were excluded from analysis	MD HHUS	4236	16	B1-2 density: MA = 80 US = 88	-	-	3.8
					B3-4 density: MA = 56 US = 88			
Berg et al. [17]	BI-RADS c-d density in at least one quadrant and at high risk; radiologist blinded to mammography and physical examination findings	MD HHUS	2809	12	77.5	-	11.2	4.2
Berg et al. [18]	BI-RADS c-d density in at least one quadrant and at high risk; radiologist blinded to mammography and physical examination findings	MD HHUS	2809	32	76	84	16	3.7
Hooley et al. [19]	BI-RADS c-d density; patients with no clinical symptoms; mammographic findings were excluded	Tech HHUS	935	3	-	-	6.5	3.2
Weigert and Steenberge [20]	BI-RADS c-d density; patients with normal mammograms; no clinical symptoms	Tech HHUS	8647	28	96.6	94.9	6.7	3.25
Weigert and Steenbergen [21]	BI-RADS c-d density; patients with normal mammograms; no clinical symptoms	Tech HHUS	10,282	24	-	96	9	2.3
Brem et al. [22]	BI-RADS c-d density; patients with normal mammograms and no clinical symptoms	Tech ABUS	15,318	30	100	72	2.6	1.9
Tagliafico et al. [23]	BI-RADS c-d density; patients with no clinical symptoms; mammography-negative; radiologist who performed ultrasound aware of negative 2D mammography and blinded to tomosynthesis	MD HHUS	3231	11 (not seen on 2D or 3D)	-	-	-	3.4 (not seen on 2D or 3D)
				23 (seen also on 3D)				7.1 (seen also on 3D)
Ohuchi et al. [24]	BI-RADS a-d density; intervention group included mammography and ultrasound on all patients; control group included mammography only; radiologists blinded to mammography and ultrasound findings	Tech HHUS	36,752	67	-	-	-	1.8
Giger et al. [25]	BI-RADS c-d density; patients with no clinical symptoms; retrospective study design	Tech ABUS	185	31	74.1	76.1		-
Wilczek et al. [26]	BI-RADS c-d density; patients with no clinical symptoms; first reader interpreted mammogram and ultrasound; second reader interpreted ultrasound only	Tech ABUS	1668	4	-	-	33.3	2.4
Weigert [27]	BI-RADS c-d density; patients with normal mammograms; no clinical symptoms; 4 year retrospective study design	Tech HHUS	Year 1: 2706	11	-	-	7.3	4
			Year 2: 3351	9	-	-	5	2.7
			Year 3: 4128	11	-	-	7.4	2.7
			Year 4: 3331	10	-	-	18.9	3
Destounis et al. [28]	BI-RADS c-d density; patients with no clinical symptoms	HHUS	5434	18	-	-	18	3.3

Note—Dashes indicate parameter was not reported in cited article. US—Ultrasound, MD—Radiologist, HHUS—Handheld ultrasound, Tech—Technologist, MA—Mammography, ABUS—Automated whole breast ultrasound.
